# Drugging the “undruggable” DNA-binding domain of STAT3

**DOI:** 10.18632/oncotarget.12181

**Published:** 2016-09-21

**Authors:** Jian-Ting Zhang, Jing-Yuan Liu

**Affiliations:** Department of Pharmacology and Toxicology and IU Simon Cancer Center, Indiana University School of Medicine, Indianapolis, IN, USA

**Keywords:** STAT3, drug discovery, DNA-binding domain, in-silico screening, small molecule inhibitor

**Figure 1:**
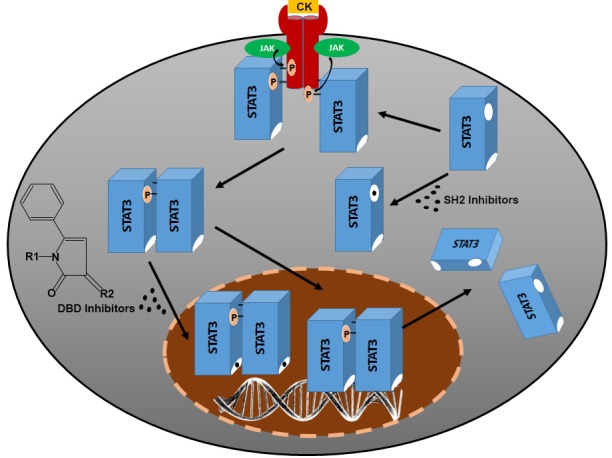
Schematic representation of JAK/STAT3 signaling pathway and direct inhibition of STAT3 activation by SH2 inhibitors and DNA-binding activity by DBD inhibitors CK, cytokine; JAK, Janus kinase; DBD, DNA-binding domain.

STAT3 (signal transducers and activators of transcription 3) is a transcription factor in the JAK/STAT signaling pathway in response to extracellular cytokines and growth factors [1]. STAT3 is constitutively activated in human malignancies and has been shown to play an important role in many facets of human cancers including oncogenesis, angiogenesis, metastasis, immune evasion, and EMT [2]. Thus, STAT3 has been a sought-after target for drug discovery. Although many STAT3 inhibitors have been identified and studied over the last decade including peptidomimetics and small molecule compounds [3], most of them target the SH2 domain, inhibiting activation and dimerization of STAT3 (Figure 1). This approach has yet to result in an FDA-approved STAT3-targeting therapeutics. Recently, STAT3-selective small molecule inhibitors targeting the DNA-binding domain (DBD) of STAT3 were identified that effectively inhibited growth and metastasis of xenograft tumors [4, 5], which may have the potential to be further developed as cancer therapeutics targeting STAT3.

DBDs of transcription factors and DNA damage repair proteins are generally considered “undruggable” with flat and similar surface areas [6, 7], which may not allow appropriate and selective inhibitor binding. Thus, these domains and sometimes the entire proteins are avoided as targets for drug discovery. In a recent study [4], Huang et al. challenged this prevailing taboo by developing an improved in-silico screening approach to eliminate potential non-selective inhibitors, in which top-scoring compounds targeting the DBD of STAT3 off the in-silico screening of a ChemDiv chemical library were docked onto the DBD of STAT1. Any compounds that were predicted to also bind to the DBD of STAT1 were eliminated due to potential lack of selectivity. This improved approach helped the authors identify a STAT3-selective hit molecule, inS3-54, which inhibited cancer cell proliferation with an IC_50_ of 3.2-5.4 μM in multiple cancer cell lines by inhibiting STAT3-dependent gene expression and inducing apoptosis [4]. However, inS3-54 had poor pharmacokinetics and was not absorbed in animal studies.

To improve the pharmacokinetic properties of inS3-54, Huang et al. [5] analyzed 69 analogues of inS3-54 and identified three analogues that were more effective than inS3-54 with IC_50_ of 1.8-5.3 μM against multiple cancer cell lines. These active analogues were not only selective to STAT3 over STAT1 as demonstrated using electrophoretic mobility shift assay, they had no effect on the survival of STAT3-null hematopoietic progenitor cells, suggesting that they are likely specific to STAT3. One of the effective analogues, inS3-54A18, was selected as a lead compound because it was soluble in a proprietary oral formulation suitable for in-vivo study and demonstrated to not only effectively inhibit cancer cell survival, but also inhibited migration and invasion of cancer cells, the in-situ DNA-binding activity of STAT3, and the growth and metastasis of xenograft tumors *in vivo* with repeated oral dosing.

Huang et al. also conducted structure-activity relationship analysis of all analogues [5], which revealed a pharmacophore, 5-phenyl-1H-pyrrol-2(3H)-ketone core structure with different functional groups (Figure 1). Compounds with R1 being p-hydroxybenzene or p-carboxybenzene had high while compounds with R1 being m-hydroxybenzene or m-carboxybenzene had low or no STAT3-inhibitory activity. The active compounds also appeared to have nitrobenzene, p-chlorobenzene, or benzenamine while inactive ones had p-methoxylbenzene as R2 group [5].

Although many inhibitors targeting the SH2 domain of STAT3 have previously been reported, most of these studies provided no direct evidence that these inhibitors indeed bind to the intended targeting site. In contrast, direct and specific binding to the DBD of STAT3 by the DBD-targeting inhibitors was validated by Huang et al. [5] using immobilized compounds and purified recombinant STAT3 proteins of various domains in a pulldown assay.

These findings on inhibitors targeting the DBD of STAT3 by Huang et al. [4, 5] not only provide potential candidates for novel anticancer drug development targeting STAT3, but also suggest that DBDs of transcription factors can be exploited as targeting sites for drug discovery and that the prevailing “undruggable” DBD of transcription factors may be druggable. Thus, targeting the DBD of oncogenic transcription factors may lead to identification of novel inhibitors that can be developed into a new class of therapeutics.
